# Menstrual Changes Following COVID-19 Vaccination: A Cross-Sectional Study

**DOI:** 10.3390/medicina60020206

**Published:** 2024-01-25

**Authors:** Nahid Ibrahim Fallatah, Bushra Omar Alrehaili, Salhah Saleh Alsulami, Abdulmohsen Hamdan Al-Zalabani

**Affiliations:** 1 Academy of Family Medicine, Ministry of Health, Madinah 42313, Saudi Arabia; mnjj.f@hotmail.com; 2 Madinah Health Cluster, Ministry of Health, Madinah 42351, Saudi Arabia; ibushra.omar@gmail.com; 3 Faculty of Medicine Rabigh, King Abdulaziz University, Jeddah 21589, Saudi Arabia; salhasolami@hotmail.com; 4 Department of Family and Community Medicine, College of Medicine, Taibah University, Madinah 42353, Saudi Arabia

**Keywords:** menstrual cycle, COVID-19, COVID-19 vaccines, cross-sectional studies, menstruation disturbances, menorrhagia

## Abstract

*Background and Objectives*: Menstrual changes, including altered cycle length and bleeding patterns, have been reported following COVID-19 vaccination. This study aimed to determine the prevalence and types of menstrual changes occurring after COVID-19 vaccination among female students and staff at a university in Saudi Arabia. *Materials and Methods*: A cross-sectional study was conducted among women aged 18–39 years who received at least one dose of a COVID-19 vaccine. Eligible participants, including university students and staff, were recruited between May 2022 and November 2022. Participants completed a questionnaire detailing their sociodemographic characteristics, general medical and reproductive history, and menstrual characteristics before and after vaccination. The prevalence of various menstrual changes (cycle length, bleeding days, flow, and mid-cycle spotting) was calculated. The demographic factors associated with menstrual changes were analyzed using chi-squared tests. *Results*: The 472 included participants had a mean age of 20.9 years, and 95.3% were unmarried. Changes in menstrual cycle characteristics after COVID-19 vaccination were reported by 54.7% of respondents overall. The most common change was in cycle length, followed by the number of menstruation days and bleeding flow. Menstrual changes were not associated with age, BMI, occupation, marital status, or medical history. Changes in intermenstrual bleeding were more frequently reported after the third dose of the Pfizer vaccine compared to the Moderna vaccine (*p* = 0.014). *Conclusions*: More than half of the recruited female students and staff reported menstrual changes following COVID-19 vaccination, with altered cycle length being the most common. The potential underlying mechanisms and implications of these menstrual alterations require further investigation. These findings provide evidence of the menstrual side effects of COVID-19 vaccines among women in Saudi Arabia.

## 1. Introduction

At the end of 2019, a novel beta-coronavirus, severe acute respiratory syndrome coronavirus 2 (SARS-CoV-2), was identified as the cause of an outbreak of pneumonia cases in Wuhan, China. The virus rapidly spread, resulting in an epidemic throughout China, followed by a global pandemic as cases arose across six continents. In February 2020, the World Health Organization (WHO) designated the disease caused by SARS-CoV-2 as COVID-19, which stands for coronavirus disease 2019 [1]. As of December 2023, over 770 million confirmed cases of COVID-19 and over 7 million deaths have been reported globally. These staggering numbers are continuously updated on the WHO and European Centre for Disease Prevention and Control (ECDC) websites [1]. While the initial COVID-19 wave in early 2020 was devastating, the rapid development and deployment of vaccines helped curb the public health crisis.

Vaccines to prevent COVID-19 infection are considered the most promising approach for curbing the pandemic and have been vigorously pursued [2]. As of 26 November 2021, the following vaccines have obtained a WHO Emergency Use Listing (EUL) [3]: Pfizer/BioNTech (New York, NY, USA) Comirnaty (31 December 2020), the SII/COVISHIELD and AstraZeneca (Cambridge, England, UK) AZD1222 vaccines (16 February 2021), the Janssen (Beerse, Belgium) Ad26.COV 2.S vaccine (12 March 2021), the Moderna (Cambridge, MA, USA) COVID-19 vaccine (mRNA 1273; 30 April 2021), the Sinopharm (Beijing, China) COVID-19 vaccine (7 May 2021), Sinovac (Beijing, China) CoronaVac (1 June 2021), and the Bharat Biotech (Hyderabad, India) BBV152 COVAXIN vaccine (3 November 2021). Four of these vaccines are approved for use in Saudi Arabia (Pfizer/BioNTech, Oxford/AstraZeneca, Janssen, and Moderna) [4]. As of November 2023, over 13 billion doses of COVID-19 vaccines have been administered worldwide [1]. Despite this progress, COVID-19 remains an urgent global health concern as new variants like Omicron emerge and vaccine equity issues persist. The COVID-19 pandemic has also highlighted many unforeseen consequences of mass vaccination campaigns, including possible effects on menstrual health, which warrant further investigation.

The menstrual cycle is the monthly phenomenon in which female hormones stimulate an ovary to release an egg, thicken the lining of the uterus to support a pregnancy, and then cause the uterus to shed this lining (through menstruation) in the absence of pregnancy [5]. The regular menstrual cycle is complex, involving the response of the endometrial lining to the dynamic hormonal reflex from the hypothalamic-pituitary-ovarian axis. Therefore, the menstrual cycle can be acutely sensitive to a variety of internal or environmental variables. These may include stress, weight fluctuations, diet, medications, inflammatory reactions, and systemic illness. Such factors have the potential to lead to changes in menstrual cyclicity, as well as characteristics such as duration, flow intensity, and accompanying symptoms like pain [6]. Some women have reported changes in their menstruation after receiving a COVID-19 vaccine [7], although the United States’ vaccine adverse event reporting system (VAERS) has recorded only a small number of menstrual cycle-related adverse events [6]. A prospective cohort study of 3959 individuals aged 18–45 years with normal cycle lengths (24–38 days) found that their menstrual cycle length had been prolonged by less than one day for both vaccine dose cycles, compared with their pre-vaccine menstrual cycles, in vaccinated women only [8].

Viable theoretical explanations for vaccine-related menstrual changes do exist, including (but not limited to) the following explanations. Menstruation itself is an inflammatory process, with the recruitment of natural killer cells, macrophages, mast cells, neutrophils, dendritic cells, and T cells playing roles in the breakdown and regeneration of the functional endometrium in each cycle. This inflammation (and/or the locally involved immunomodulatory molecules such as cytokines and chemokines) could be influenced by the systemic immune response to the COVID-19 vaccine [6]. In addition, the angiotensin-converting enzyme II (ACE-2) receptor, the target of the spike protein of COVID-19 viruses in a range of biological tissues, is expressed in the uterus and is thought to play a functional role in the differentiation of endometrial fibroblasts into decidual stromal cells in the secretory phase prior to menstruation [6].

An association between menstrual changes and COVID-19 vaccines exists but has not been investigated in a rigorous or systematic manner. Therefore, this study investigated the connection between COVID-19 vaccination and changes in menstruation to provide an accurate guide for patients and healthcare providers. The findings may have broader implications for reproductive health monitoring and care provision globally in response to an unprecedented mass immunization effort against a novel pathogen.

## 2. Materials and Methods

This cross-sectional analytical study was carried out at Taibah University in Al-Madinah City. Al-Madinah City is the fourth largest region by population in Saudi Arabia, with a population of 1,152,991 persons [9]. Taibah University was established in the year 2003 and includes 28 colleges and 1 institute. The university has 1409 faculty members, in addition to 1606 teaching assistants, lecturers, and language teachers. The number of students was estimated to be 69,210 male and female students in the academic year 1439–1440 AH [10].

All regular female academic staff and students of Taibah University during the study period were eligible to participate in the study. The inclusion criteria were as follows: women aged between 18 and 39 years who received the first dose or a complete cycle of a COVID-19 vaccine, regardless of vaccine type, and who reported having a regular menstrual cycle before vaccination. Inclusion was not limited to individuals of Saudi nationality. Conversely, women having an irregular menstrual cycle before vaccination, those taking any kind of hormonal therapy, including combined oral contraceptives, and those diagnosed with polycystic ovary syndrome (PCOS) were excluded.

Epi Info™ version 7.2.5.0 software (CDC, Atlanta, GA, USA) was used to calculate the sample size [11] for this study, based on the following considerations: a 50% prevalence of menstrual irregularities after COVID-19 vaccination (as no study has been conducted in Medina thus far), a confidence limit of 95%, and a margin of error of 5%. The required sample size was found to be 331 participants. Upon accounting for non-responders and missing data, the sample size increased to 350 participants.

A questionnaire was designed by 4 women’s health experts after reviewing the relevant literature [12,13,14,15]. It included 4 parts: (1) sociodemographic characteristics: age, marital status, occupation, and educational level; (2) general medical and reproductive history and lifestyle information: height, weight, chronic medical conditions, pregnancy, breastfeeding, contraception use, and smoking status; (3) menstrual patterns; and (4) self-reported menstrual irregularities after receiving a COVID-19 vaccine: changes in the schedule (time between menses or the length of the cycle) and duration (days of menstrual bleeding) of menses, menstrual flow (heavier or lighter bleeding), and intermittent bleeding (spotting). Questions regarding the type of vaccine and the number of doses received were also included. The questionnaire was tested on 10 women prior to its implementation in the study area to assess its applicability and validity, and necessary changes to the questionnaire were made. The 10 pretest women were excluded from the study.

Data were collected between May 2022 and November 2022. A stratified, proportional-to-size random sample of staff and students were invited by email to participate and fill out an online survey. Informed consent was obtained from the participants prior to data collection. Ethical approval was obtained from the Institutional Review Board of the General Directorate of Health Affairs in Al Madinah City (approval number 022-47, on 29 May 2022). Approval for data collection was obtained from the Academic Affairs Department of Taibah University, following ethical approval. Data privacy and confidentiality were assured for all collected data, and the data were used only for study purposes.

### Statistical Analysis

The collected survey data were systematically analyzed using the Statistical Package for Social Sciences (SPSS), version 21.0 (SPSS, Chicago, IL, USA) [16]. Categorical data were described using frequency and percentage as n (%). Quantitative variables such as age and body mass index were described using mean and standard deviation (mean ± SD). The prevalence of various menstrual irregularities after COVID-19 vaccination and its 95% confidence interval were calculated. Cross-tabulations and chi-squared tests were utilized to examine the potential associations between experiencing menstrual changes post-vaccination and the participants’ demographic and health characteristics. Differences in the specific menstrual irregularities that were reported following the first, second, and third doses of various COVID-19 vaccine types were compared using chi-squared and Fisher’s exact tests. All tests were two-sided and a *p*-value < 0.05 was considered statistically significant.

## 3. Results

A total of 539 women were screened for inclusion in the study. In total, 11 women were excluded because they had been pregnant or breastfeeding in the previous year and 56 were excluded because they had a history of PCOS. The survey data of the 472 people who met the inclusion criteria and had completed the survey were analyzed. The majority (95.3%) were unmarried, with a mean age of 20.9 (4.4) years, and a level of education of high school or below (71.0%). In terms of health, roughly half of the respondents (53.4%) were of normal weight, based on their height and weight measurements, while 20.8% reported a history of disease. More than half of the participants (60.8%) had previously had a COVID-19 virus infection. Other characteristics are shown in Table 1.

### 3.1. Menstrual Changes after the First, Second, and Third Vaccine Doses

**Figure 1:**
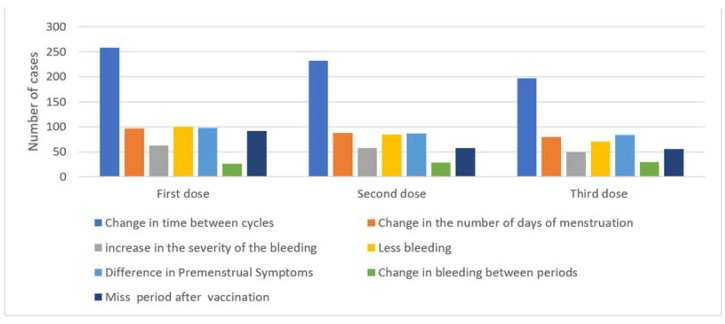
Menstrual changes after COVID-19 vaccination.

Figure 1 illustrates the common menstrual changes observed following the first, second, and third vaccine doses. Changes in the length of time between menstrual cycles were the most common change reported among recipients across all 3 doses, while a change in the number of menstruation days was the second most common change. Conversely, among recipients across all 3 doses, a change in intermenstrual bleeding was the least commonly observed change.

### 3.2. Menstrual Cycle Characteristics of the Participants

Table 2 displays the participants’ menstrual cycle characteristics. The average cycle length was 24–38 days among 73.1% (*n* = 345) of respondents. Participants with heavy menstrual flow comprised 8.7% of the study sample, and approximately 14.6% of the women reported irregular or longer bleeding days. Intermenstrual bleeding was experienced by 11.2% of women, and 45.6% of all women had used period-tracking apps on their smart devices (phones or tablets) to keep track of their cycles.

### 3.3. Association between Menstrual Cycle Changes Following COVID-19 Vaccination and Participant Characteristics

The most encountered menstrual changes were noticed after the first dose of vaccine in 258 (54.7%) participants. Menstrual cycle changes did not differ according to marital status, body mass index (BMI), occupation, and medical history. Table 3 displays the specific changes.

### 3.4. Menstrual Cycle Changes According to Vaccine Brand

The most commonly administered vaccines for the first and second doses were Pfizer and AstraZeneca. Following receipt of the first vaccine dose, 90 (21.5%) Pfizer recipients reported changes in PMS, and 11 (22.4%) of AstraZeneca recipients reported menorrhagia. In addition, 75 (19.2%) Pfizer recipients reported a change in PMS after the second dose. The most common change in AstraZeneca second-dose recipients was prolonged menstruation, which occurred in 14 (27.5%) participants. Compared to those who received the Moderna vaccine, third-dose recipients of the Pfizer vaccine reported higher levels of intermenstrual hemorrhage (*p* = 0.014). Due to the small number of participants, additional vaccination types were not analyzed. Table 4 includes details regarding menstrual changes according to the different vaccine types.

## 4. Discussion

The present study investigated the menstrual cycle changes that occur after COVID-19 vaccination. A sample of 472 women with a mean age of 21 years was assessed. The results demonstrated that a significant proportion of participants experienced changes in their menstrual cycles following COVID-19 vaccination. The association between COVID-19 vaccination and menstrual cycle changes has previously been reported in several studies [1,2,17,18]. The most common change reported in our current survey was an alteration in the time between cycles, which is in line with the previously published literature [1,2]. For instance, a study by Edelman et al. showed that 66.3% of women reported menstrual changes following vaccination [3]. An American cohort showed that vaccinated women’s menstrual cycles were one day longer than those of non-vaccinated women [12]. With the remaining evidence, the vaccine was proposed as a factor contributing to menstrual changes rather than the pandemic effect. A large population-based study of women who used menstruation-tracking mobile apps found no evidence of ovulatory or menstrual changes during the pandemic [19]. This is consistent with our findings, which showed that nearly half of the participants used smartphone-based menstruation trackers. Furthermore, they had average-length cycles, moderate bleeding flows, and minimal cycle variability prior to vaccination.

Menstrual cycle changes were initially listed as a common side effect of vaccination. Heavy menstrual bleeding was reported by the yellow card system in the United Kingdom among Pfizer and Moderna recipients, although most of these events were minor and temporary in nature [4,20]. In Saudi Arabia, Pfizer-BioNTech and Oxford-AstraZeneca are the most frequently used vaccine types. The current survey found no differences in menstrual cycle changes among the recipients of these vaccines. However, Pfizer recipients reported intermenstrual bleeding more commonly than Moderna recipients.

Menstrual disturbances following COVID-19 vaccination can be explained by several mechanisms. For example, the vaccine may initially induce a systemic reaction [21] and, in a sizable proportion of women, may cause an acute illness that may alter the hormone balance in the hypothalamic-pituitary-gonadal axis [22,23]. These hormonal changes can lead to temporary disruptions in menstrual patterns and flow. This argument has been made for other vaccines such as HPV-4 and HPV-9 as they have been linked to premature ovarian insufficiency and irregular menstruation [24]. These menstrual changes were also reported in several studies among women post-COVID-19 [25]. In addition to hormones, the menstrual cycle involves a complicated interaction between endometrial tissues and the recruitment of a variety of immune cell populations, which may be altered by COVID-19 vaccine components [26,27]. Additionally, it is important to consider that the psychological stress associated with vaccination or illness could also play a role in causing menstrual irregularities, rather than vaccination. However, a cohort study involving a large group of participants did not identify any widespread changes in menstrual cycles in the population during the pandemic [19].

An important question raised by the evidence of menstrual changes following COVID-19 vaccination is whether these effects are transient or could have longer-lasting impacts. The duration of post-vaccination menstrual irregularities warrants further longitudinal investigation. In this cross-sectional study, the timeframe for menstrual changes was not examined. Data were collected at a single point, so it remains unclear if the alterations persisted over subsequent cycles or resolved themselves quickly. Following participants over multiple months via diaries or tracking apps could elucidate the duration of menstrual disruptions like altered cycle lengths, flow changes, or spotting. This is clinically relevant, as longer-term menstrual disturbances may signal deeper impacts on ovarian function and fertility. If confirmed as a temporary side effect, counseling women that menstrual irregularities quickly self-resolve may prevent anxiety. However, if this is demonstrated to be a more persistent issue, screening for reproductive health impacts like altered hormonal levels, ovarian reserves, or egg quality may become necessary after vaccination. The potential for lasting fertility changes is an understandable concern for many young women that must be either conclusively ruled out or addressed through counseling and awareness. Rigorously designed, large-scale longitudinal studies are needed to provide definitive evidence on whether COVID-19 vaccination leads to short-term menstrual flux or sustains changes over time, with possible reproductive effects. Following cohorts of vaccinated women closely through meticulous data collection over successive months would offer vital insights.

Even if menstrual changes are confirmed as short-lived side effects, the experience of one’s usual cycle altering can cause stress, anxiety, confusion, and diminished quality of life. Women experiencing unfamiliar breakthrough bleeding, prolonged cycles, or sudden shifts in flow or timing after vaccination may become concerned about their health. Uncertainty regarding the cause and duration of menstrual disturbances can compound frustration. Some women may feel unsupported or dismissed if menstrual fluctuations are minimized as transient or as expected vaccine outcomes, without acknowledgment of their lived experience. Providing counseling services and creating support resources for women experiencing menstrual changes post-vaccination, no matter how physiologically minor, demonstrates empathy and prevents isolation. Active listening and the validation of women’s experiences, paired with evidence-based education on the expected timelines and outcomes, can alleviate uncertainty. It is also important to screen for and address the resulting mood changes or other mental health impacts. In addition, preparing women ahead of time for the possibility of menstrual effects may ease anxiety. The psychosocial influence of such changes on women’s health-related quality of life should be assessed and supported through resources aimed at fostering coping mechanisms, resilience, and knowledge.

The main strength of this study is its inclusion of a homogeneous sample of unmarried and young women with a school-level education. Additionally, nearly half of the sample currently uses period-tracking apps to monitor their menstrual cycles. Women with PCOS and pregnant and breastfeeding women were excluded from the study. This decision was made because most women, especially those who breastfeed, experience amenorrhea or menstrual irregularities after giving birth. Additionally, we investigated how the frequency, length, and number of the ensuing menstrual cycles changed, stratifying the data according to the vaccine type for analysis. Data for the first, second, and third doses were meticulously collected and analyzed. Lastly, the study included comprehensive data from the local population in Saudi Arabia. The country had several waves of COVID-19 [28] and adopted several preventive measures, including vaccination [21]. However, few studies addressed COVID-19 vaccination side effects among the Saudi population.

Conversely, it is important to note that the study has several limitations worth considering. First, the study relied on self-reported data, which may be subject to recall bias and social desirability bias. Second, the cross-sectional design of the study cannot establish causality between COVID-19 vaccination and menstrual changes. Third, due to the limited geographic area of sampling, generalizability may be an issue. Finally, it is essential to acknowledge that the menstrual cycle involves complex hormonal interactions that can be affected by several factors, including pandemic-related stress. Future studies should aim to address these limitations. Additionally, further investigations are also required to thoroughly assess and understand the potential mechanisms by which COVID-19 vaccination affects the menstrual cycle.

Conversely, it is important to note that the study has several limitations that must be acknowledged when interpreting the findings. First, a reliance on retrospective self-reported survey data may introduce recall bias or social desirability bias regarding menstrual symptoms. Utilizing prospective menstrual diaries completed in real time could improve accuracy. Second, a cross-sectional study design captures menstrual function at only a single time point, preventing causal inferences about vaccination effects over time. Longitudinal cohorts are needed to confirm temporality. Third, convenience sampling from a limited geographic region may not represent the wider population, restricting generalizability. Multi-center studies with probability sampling would enhance representation. Fourth, potential confounding factors like stress, BMI changes, or the use of medications were not addressed. Adjusting for confounders in future analyses could isolate an independent association with vaccination. Finally, the complex neuroendocrine regulation of menstrual cyclicity can be impacted by numerous influences beyond just COVID-19 immunization. While unlikely, given the respondents’ pre-pandemic regularity of cycle, other pandemic-related biopsychosocial effects cannot be ruled out. Control groups will help separate vaccine versus pandemic impacts on menstrual health. Overall, this exploratory study provides guiding evidence that warrants verification through more rigorous randomized controlled trials and longitudinal designs controlling for potential confounders. Examining in detail the biological mechanisms and triangulating self-reported data with objective clinical measures could also help establish a better understanding and refine clinical recommendations.

## 5. Conclusions

In conclusion, the present study provides further evidence of a potential link between COVID-19 vaccination and menstrual changes among young women. The high prevalence of altered cycle length, flow, timing, and symptoms after vaccination warrants attention from both researchers and clinicians. These findings have important implications for women’s health and highlight the need for further high-quality, longitudinal research to confirm the associations and elucidate the underlying biological mechanisms. Additional studies should follow diverse cohorts of women over time to characterize the duration and downstream impacts of menstrual irregularities post-vaccination. Healthcare providers, especially those in the reproductive health fields, should be made aware of these potential side effects through enhanced public health messaging and education campaigns. Clinical recommendations and talking points should be developed to guide providers in having empathetic discussions with patients about expected timelines and address any concerns or anxiety. Appropriate screening, counseling, education, and symptom management support need to be made readily available to women who experience concerning menstrual changes after COVID-19 vaccination. Open communication and active listening will be key.

## Figures and Tables

**Table 1:** Sociodemographic characteristics of the study sample (*n* = 472).

Variable	Parameters	Frequency (%)
Age	Mean (SD)	20.9 (4.4) *
Marital status	Unmarried (single, widowed, divorced)	450 (95.3%)
	Married	22 (4.7%)
Education	High-school level or below	335 (71.0%)
	University	129 (27.3%)
	Postgraduate	8 (1.7%)
Occupation	Working	18 (3.8%)
	Student	454 (96.2%)
BMI classes	Underweight	112 (23.7%)
	Normal weight	252 (53.4%)
	Overweight	67 (14.2%)
	Obesity class 1	28 (5.9%)
	Obesity class 2	7 (1.5%)
	Obesity class 3	6 (1.3%)
Medical history	I don’t have any diseases	374 (79.2%)
	Yes	99 (20.8%)
Type of disease	Diabetes mellitus	7 (1.5%)
	Hypertension	7 (1.5%)
	Thyroid diseases	9 (1.9%)
	Anemia	73 (15.5%)
Smoking history	I have never smoked in my life	442 (93.6%)
	I used to smoke before, but I stopped smoking	11 (2.3%)
	Yes, I currently smoke less than 10 cigarettes a day	4 (0.8%)
	Yes, I currently smoke non-daily	15 (3.2%)
Have you had a COVID-19 infection?	Yes	185 (39.2%)
	No	287 (60.8%)
How many times have you had a COVID-19 infection?	Once	154 (32.6%)
	Twice	27 (5.7%)
	Three times	2 (0.4%)
	Never	289 (61.2%)

* Mean (SD).

**Table 2:** Menstrual cycle characteristics of the participants.

Variable	Parameters	Frequency (%) *n* = 472
Cycle length variability	≤8 days	306 (64.8%)
	>8 days	166 (35.2%)
Cycle length	Less than 24 days	96 (20.3%)
	Between 24 and 38 days	345 (73.1%)
	More than 38 days	31 (6.6%)
Menstrual flow	Low (less than 5 mL)	49 (10.4%)
	Moderate (5–80 mL)	382 (91.3%)
	Severe (more than 80 mL/clots)	41 (8.7%)
Bleeding days	Less than 8 days	403 (85.4%)
	More than 8 days	23 (4.9%)
	Too irregular	46 (9.7%)
Mid-cycle spotting	Yes	53 (11.2%)
	No	419 (88.8%)
PMS symptoms	Yes	447(94.7%)
	No	25 (5.3%)
Do you use a smart device application to monitor your menstrual cycle?	Yes	215 (45.6%)
	No	257 (54.4%)

**Table 3:** Association between menstrual cycle changes and participant characteristics.

Variables	Reporting Cycle Change after First Dose 258 (54.7%)		Reporting Cycle Change after Second Dose 232 (49.6%)		Reporting Cycle Change after Third Dose 197 (52.3%)	
	Yes	*p*-Value	Yes	*p*-Value	Yes	*p*-Value *
Marital Status						
Single	243 (54.6%)	0.99	218 (49.4%)	0.531	182 (51.3%)	0.286
Married	12 (54.5%)		10 (45.5%)		12 (63.2%)	
Occupation						
Employee	13 (72.2%)	0.15	8 (44.4%)	0.657	8 (61.5%)	0.58
Student	245 (54%)		224 (49.8%)		189 (51.9%)	
BMI Classes						
Non-overweight/non-obese	197 (54.3%)	0.814	179 (49.9%)	0.755	141 (49.8%)	0.122
Overweight/obese	60 (55.6%)		52 (48.1%)		55 (59.1%)	
Medical History						
Healthy	208 (55.6%)	0.427	184 (49.7%)	0.91	159 (52.8%)	0.377
Diagnosed with a medical problem	50 (51%)		48 (49.0%)		38 (50.0%)	

* Chi-squared and Fisher’s exact tests were used, and a *p*-value of < 0.05 was considered statistically significant.

**Table 4:** Menstrual cycle changes according to different types of COVID-19 vaccination.

	Prolonged Menstruation		Menorrhagia		Change in PMS		Intermenstrual Bleeding		Missed Period	
	*n* (%)	*p*-Value	*n* (%)	*p*-Value	*n* (%)	*p*-Value	*n* (%)	*p*-Value	*n* (%)	*p*-Value *
First dose										
Pfizer	83 (19.9%)	0.341	52 (12.4%)	0.206	90 (21.5%)	0.563	24 (5.7%)	0.687	86 (20.6%)	0.344
AstraZeneca	14 (28.6%)		11 (22.4%)		8 (16.3%)		1 (2%)		5 (10.2%)	
Second dose										
Pfizer	69 (17.7%)	0.417	49 (12.6%)	0.458	75 (19.2%)	0.58	25 (6.4%)	0.598	44 (11.3%)	0.338
AstraZeneca	14 (27.5%)		8 (15.7%)		6 (11.8%)		2 (3.9%)		10 (19.6%)	
Third dose										
Pfizer	63 (20.2%)	0.327	42 (13.5%)	0.98	69 (22.1%)	0.671	26 (8.3%)	0.014	48 (15.4%)	0.103
Moderna	14 (29.8%)		6 (12.8%)		10 (21.3%)		0 (0%)		4 (8.5%)	

* Chi-squared and Fisher’s exact tests were used, and a *p*-value of < 0.05 was considered statistically significant.

## Data Availability

The data presented in this study are available on request from the corresponding author. The data are not publicly available due to restrictions from the institution.
